# Supplementary material for: Unsupervised Decoding of Long-Term, Naturalistic Human Neural Recordings with Automated Video and Audio Annotations
Source: Front Hum Neurosci. 2016 Apr 21;10:165. doi: 10.3389/fnhum.2016.00165 (PMC4838634; doi:10.3389/fnhum.2016.00165)
Supplement: Supplementary file 5 [file Presentation1.PDF]

# *Supplementary Material*: Unsupervised decoding of long-term, naturalistic human neural recordings with automated video and audio annotations

**Nancy X. R. Wang*, Jared D. Olson, Jeffrey G. Ojemann, Rajesh P. N. Rao, and Bingni W. Brunton**

*Correspondence:
Author Name: Nancy X.R. Wang
wangnxr@uw.edu

**Video S1.** Cartoon of infrared video showing Subject 6 apparently asleep at 11:50PM on day 6 post implant, corresponding to the data shown in Fig. 4. The hours between 10:00PM and 7:00AM were clustered as sleep by our method.

**Video S2.** Cartoon of video showing Subject 6 taking a nap during the day time at 11:24am on day 6 (at time marked by triangle in Fig. 4). The subject's family member is waiting by the subject's side and the video is in color, indicating daytime. This rest period is picked up as a predominant rest cluster in our decoding method.

**Video S3.** Cartoon of video showing Subject 6 moving around on day 6 post implant (at time marked by square in Fig. 4). The subject had recently woken up and was moving around for adjustment at 12:58pm. There was no audible speech at the same time; for patient privacy, we cannot include sound in this supplemental video. This time period is mostly clustered as movement by our method.

**Video S4.** Cartoon of video showing Subject 6 engaging in active conversation at 5:10pm on day 6 post implant (at time marked by circle in Fig. 4). The subject is talking and listening; for patient privacy, we cannot include the sound of the video and the face is blurred. This time period is clustered mainly as speaking by our method.

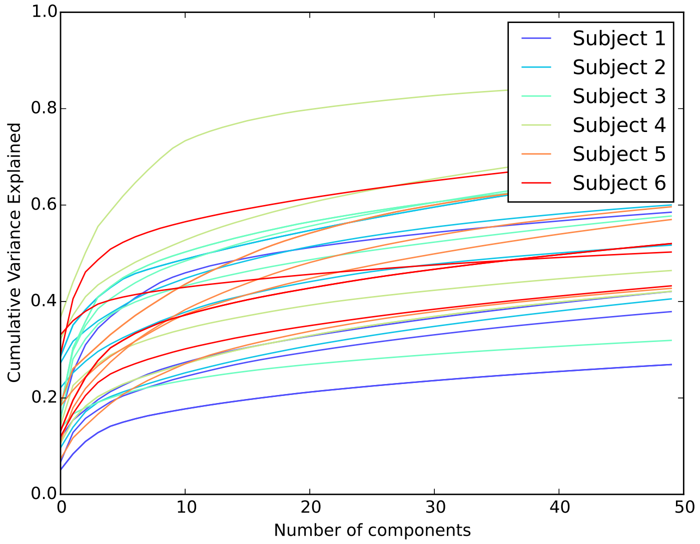


**Figure S1:** Cumulative variance explained by number of principal components (from PCA) shows that there is a wide variation across subjects and days. Although more components may be more accurate in terms of percentage of variance explained, k-means does not work well in very high dimensional space, so we chose to use 50-dimensional PCA features space for clustering. At 50 components, a large majority of days have at least 0.4 accumulated variance.

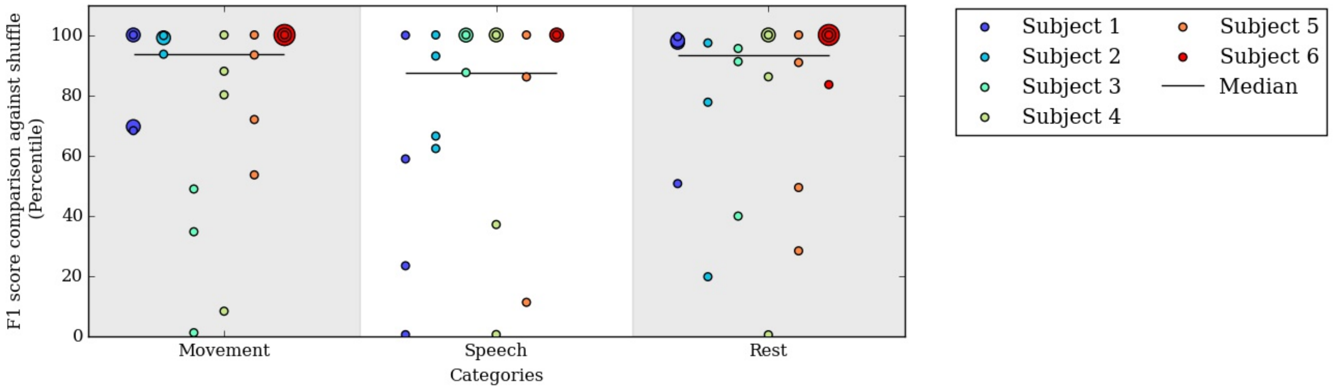


**Figure S2:** Percentile of the F1 score of our algorithm compared to manually annotated labels. Decoding at level 2 shows far worse performance than level 3, which is shown in the main text Figure 5. Each colored dot indicates the percentile of one day for a subject for a behavioural category.

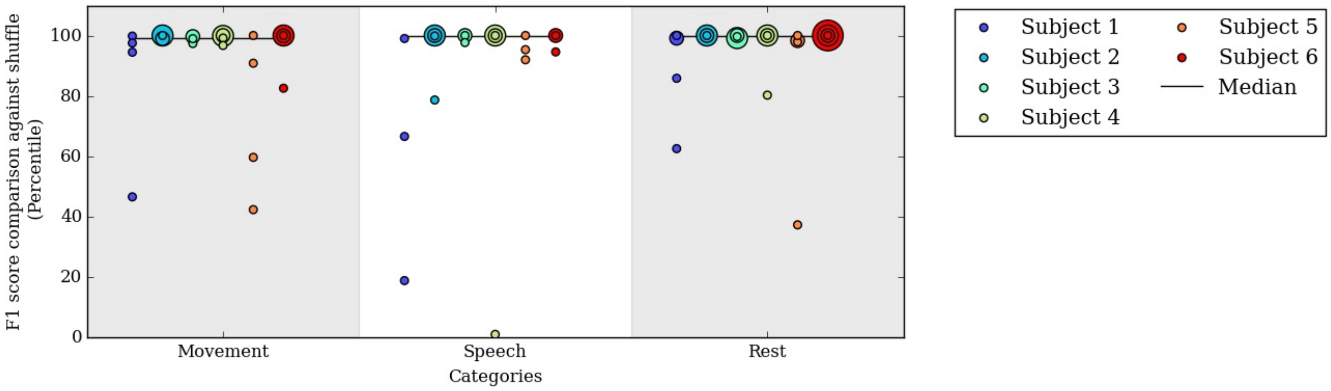


**Figure S3:** Percentile of the F1 score of our algorithm compared to manually annotated labels. Decoding at level 4 shows comparable performance than level 3, which is shown in the main text Figure 5. However, the number of time points belonging to each cluster is rather low, as shown in the level 4 graph of Figure 4 in the main text. Each colored dot indicates the percentile of one day for a subject for a behavioural category.

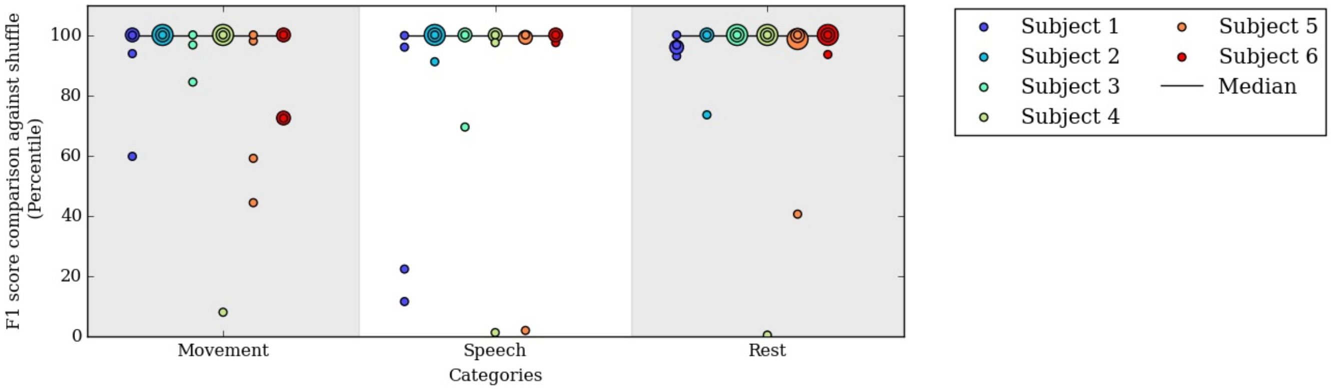


**Figure S4:** Percentile of the F1 score of our algorithm using features containing frequencies up to 105 Hz as compared to manually annotated labels. Decoding at level 3 using spectral frequencies up to 105Hz shows comparable performance as compared to level 3 using power from frequencies below 52Hz as shown in the main text Figure 5. However, as shown in the table S2, the accuracy using the high powers is worse than than without (as shown in the main text Table 1).

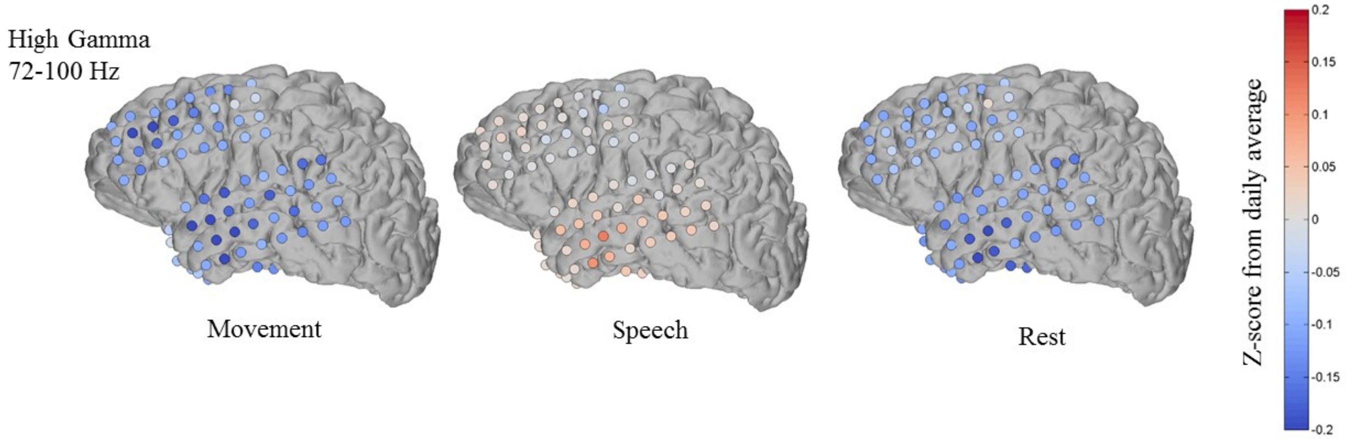


**Figure S5:** Features discovered by automated brain decoding using high gamma power band for Subject 1 day 6 post implant did not show localized high power levels during movement, as may be suggested by motor mapping experiments. However, there is some high power activity in the temporal region that may warrant further studies.

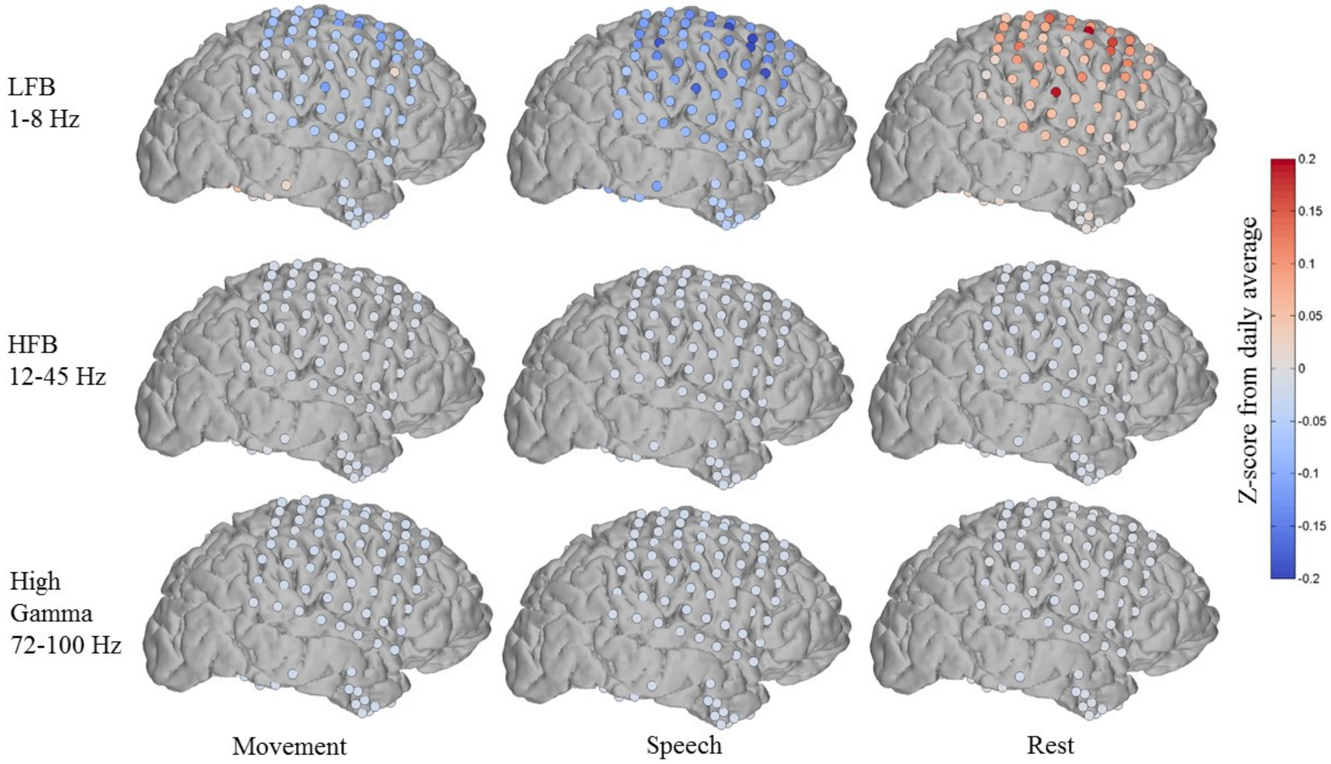


**Figure S6:** Features discovered by automated brain decoding using high gamma power band for a sample day of Subject 3 showing high power during rest in LFB and lower power during movement and speech. However, no localized activity changes can be detected.

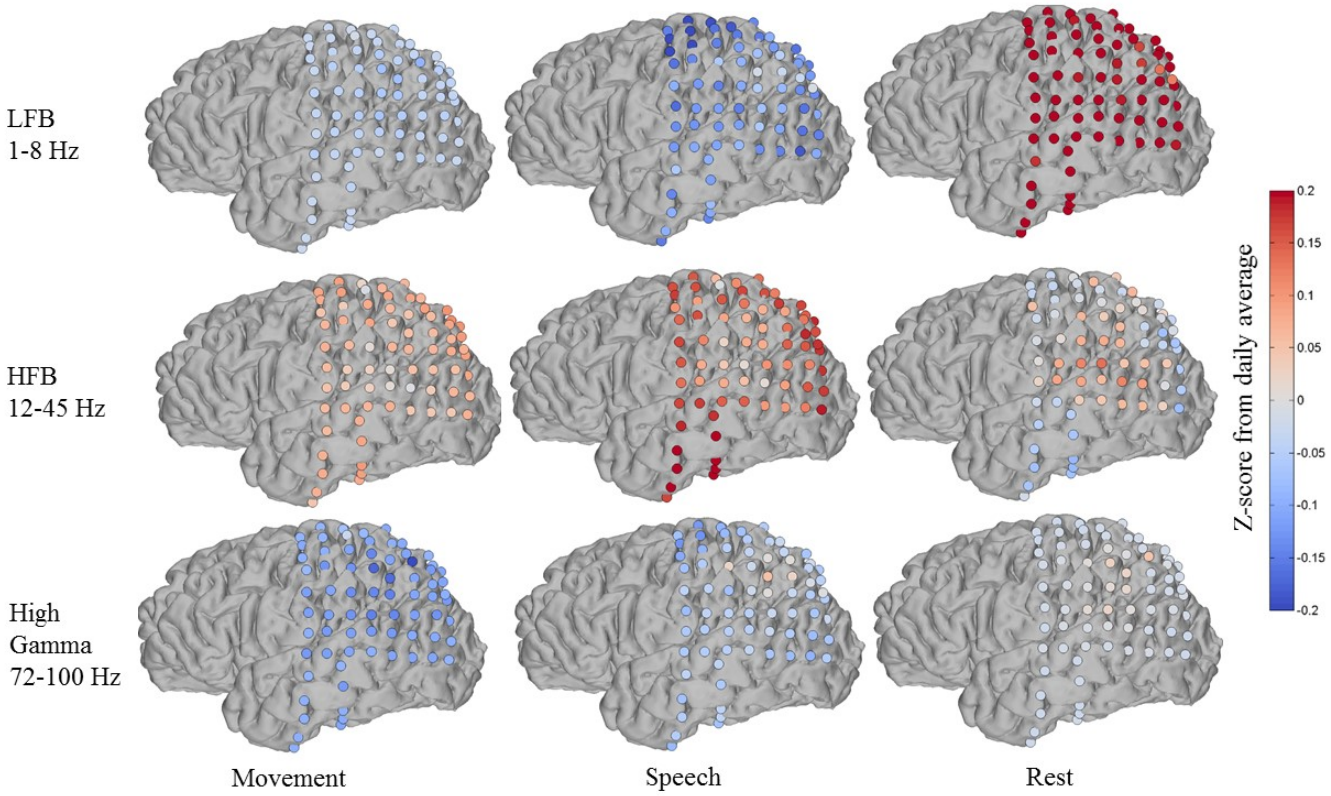


**Figure S7:** Features discovered by automated brain decoding using high gamma power band for a sample day of Subject 4 showing high power during rest in LFB and lower power during movement and speech. This activity level is flipped to high for movement and speech but lowered during rest in HFB. No significant pattern is detected for high gamma.

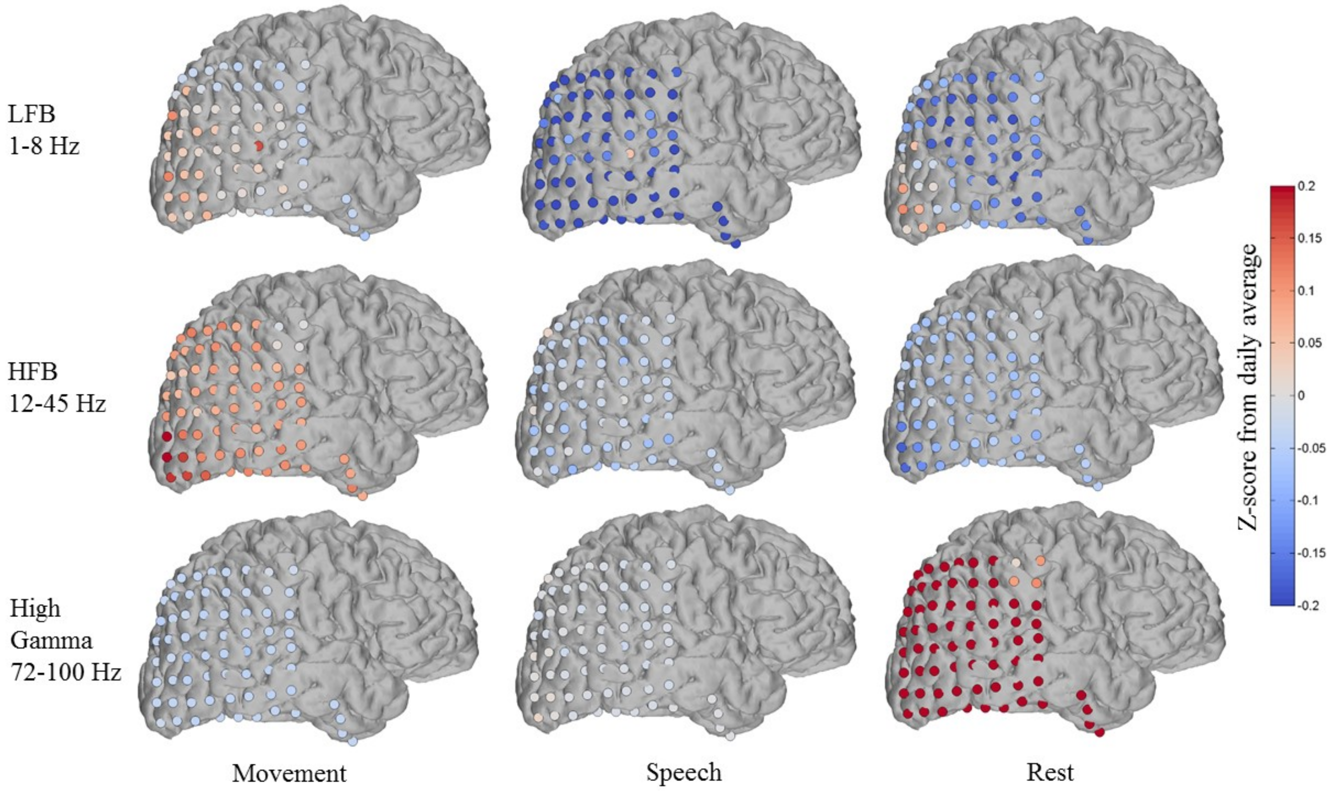


**Figure S8:** Features discovered by automated brain decoding using high gamma power band for a sample day of Subject 5 showing power changes in the visual cortex. This may be due to high correlations between movement, speech and visual sensing.

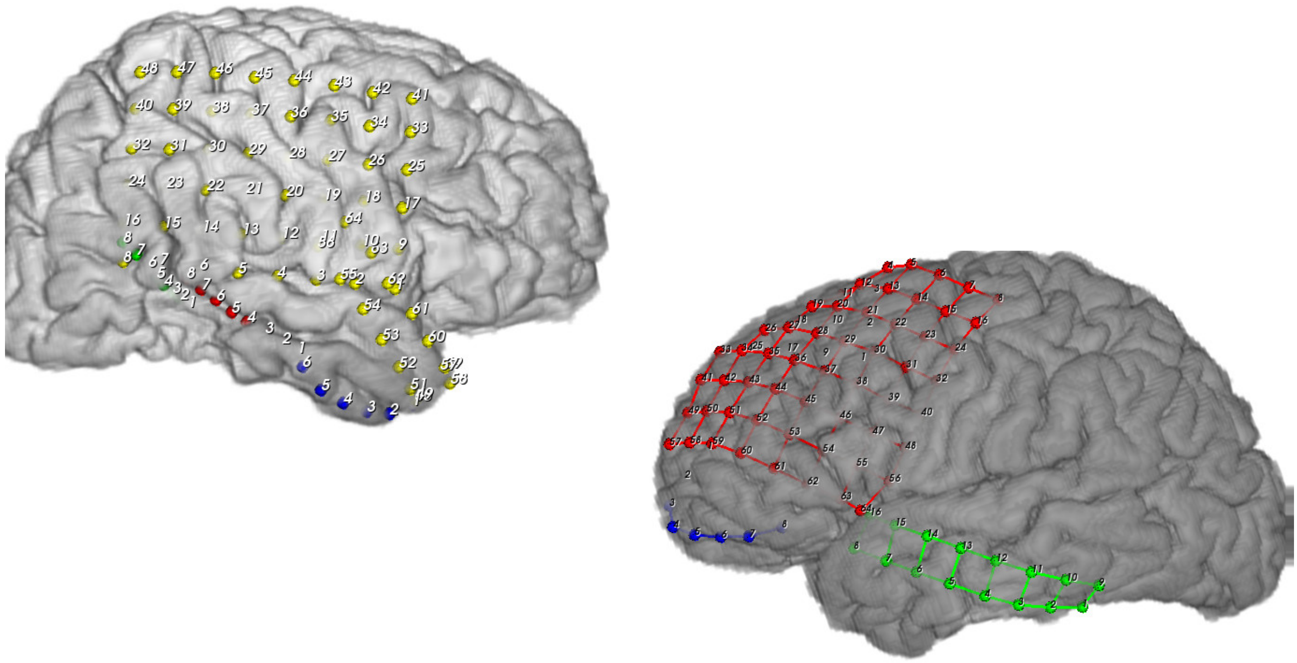

**Figure S9:** Clinical electrode maps are shown for subject 6 (left) and subject 2 (right). Electrode location reconstruction information was not available for these two subjects, so mapping of cluster centroids was not possible.

| | Movement | | | | Speech | | | | Rest | | | |
|---|---|---|---|---|---|---|---|---|---|---|---|---|
| | F1 | R_F1 | R_std | Pctl | F1 | R_F1 | R_std | Pctl | F1 | R_F1 | R_std | Pctl |
| S1 D3 | 0.58 | 0.36 | 5.67 | 100.00 | 0.05 | 26.28 | 6.02 | 0.00 | 0.68 | 0.53 | 3.84 | 100.00 |
| D4 | 0.60 | 0.41 | 6.42 | 99.90 | 0.16 | 0.31 | 7.95 | 2.70 | 0.82 | 0.68 | 3.35 | 100.00 |
| D5 | 0.53 | 0.39 | 5.28 | 99.60 | 0.58 | 0.51 | 3.73 | 96.00 | 0.40 | 0.29 | 5.97 | 96.40 |
| D6 | 0.27 | 0.27 | 4.88 | 50.20 | 0.42 | 0.31 | 6.12 | 96.60 | 0.55 | 0.51 | 3.64 | 86.25 |
| S2 D3 | 0.39 | 0.36 | 1.55 | 94.05 | 0.68 | 0.56 | 2.15 | 100.00 | 0.35 | 0.30 | 2.49 | 98.65 |
| D4 | —- | —- | —- | —- | 0.70 | 0.69 | 1.44 | 61.95 | —- | —- | —- | —- |
| D5 | 0.56 | 0.47 | 3.48 | 99.75 | 0.56 | 0.36 | 4.53 | 100.00 | 0.68 | 0.36 | 4.68 | 100.00 |
| D6 | 0.63 | 0.51 | 2.89 | 100.00 | 0.71 | 0.61 | 2.59 | 99.90 | 0.47 | 0.25 | 5.52 | 100.00 |
| S3 D3 | —- | —- | —- | —- | —- | —- | —- | —- | —- | —- | —- | —- |
| D4 | 0.48 | 0.39 | 4.98 | 96.15 | 0.79 | 0.44 | 4.99 | 100.00 | 0.80 | 0.54 | 3.89 | 100.00 |
| D5 | 0.34 | 0.26 | 5.23 | 92.85 | 0.67 | 0.47 | 3.69 | 100.00 | 0.74 | 0.39 | 4.01 | 100.00 |
| D6 | 0.58 | 0.55 | 3.06 | 80.95 | 0.52 | 0.44 | 4.04 | 98.05 | 0.51 | 0.34 | 4.93 | 100.00 |
| S4 D3 | 0.59 | 0.51 | 3.05 | 99.45 | 0.58 | 0.31 | 4.46 | 100.00 | 0.70 | 0.48 | 3.22 | 100.00 |
| D4 | 0.65 | 0.57 | 2.85 | 99.85 | 0.40 | 0.30 | 4.76 | 98.25 | 0.61 | 0.40 | 3.94 | 100.00 |
| D5 | 0.64 | 0.57 | 3.19 | 98.45 | 0.34 | 0.47 | 3.98 | 0.00 | 0.21 | 0.39 | 4.55 | 0.00 |
| D6 | 0.54 | 0.30 | 4.95 | 100.00 | 0.67 | 0.36 | 5.16 | 100.00 | 0.22 | 0.15 | 3.38 | 98.05 |
| S5 D3 | 0.43 | 0.44 | 4.36 | 45.20 | 0.33 | 0.31 | 3.86 | 70.00 | 0.64 | 0.61 | 3.11 | 75.35 |
| D4 | 0.44 | 0.34 | 7.25 | 90.30 | —- | —- | —- | —- | 0.78 | 0.78 | 2.52 | 52.65 |
| D5 | 0.31 | 0.26 | 8.69 | 70.95 | 0.38 | 0.36 | 7.48 | 57.65 | 0.59 | 0.59 | 4.99 | 52.60 |
| D6 | 0.64 | 0.37 | 5.12 | 100.00 | 0.73 | 0.36 | 5.35 | 100.00 | 0.83 | 0.60 | 3.28 | 100.00 |
| S6 D3 | 0.71 | 0.68 | 1.78 | 93.25 | 0.58 | 0.49 | 4.10 | 97.90 | 0.57 | 0.42 | 4.80 | 100.00 |
| D4 | 0.69 | 0.37 | 5.02 | 100.00 | —- | —- | —- | —- | 0.86 | 0.47 | 4.40 | 100.00 |
| D5 | 0.29 | 0.32 | 3.37 | 23.95 | 0.67 | 0.21 | 6.91 | 100.00 | 0.79 | 0.66 | 2.51 | 100.00 |
| D6 | 0.71 | 0.49 | 3.83 | 100.00 | 0.76 | 0.49 | 3.81 | 100.00 | 0.92 | 0.34 | 5.67 | 100.00 |

**Table S1**. F1 scores and percentiles as assessed by comparison of level 3 automated cluster annotation to manual annotations shown for each of the 4 days analyzed for each subject. The true F1 scores are compared to randomly shuffled F1 scores. Acc = Accuracy; R_acc = mean of random shuffle accuracy; R_std = standard deviation of random shuffle accuracy; Pctl = percentile of accuracy score within random shuffles; S = Subject; D = Day; — = Not enough manual labels were collected for . These F1 scores correspond to the same data shown as percentiles against random shuffle in Fig. 5.

| **Movement** | Acc | F1 | Spc | Sen/Rec | Prc |
|---|---|---|---|---|---|
| Subject 1 | 60.99 | 0.52 | 72.45 | 49.12 | 59.94 |
| Subject 2 | 59.95 | 0.67 | 76.37 | 56.25 | 85.59 |
| Subject 3 | 61.57 | 0.44 | 72.99 | 42.64 | 49.01 |
| Subject 4 | 57.00 | 0.59 | 52.16 | 61.90 | 56.05 |
| Subject 5 | 55.16 | 0.40 | 61.91 | 48.58 | 50.57 |
| Subject 6 | 68.96 | 0.65 | 68.39 | 61.86 | 73.83 |
| **Speech** | Acc | F1 | Spc | Sen/Rec | Prc |
| Subject 1 | 50.18 | 0.42 | 46.75 | 57.21 | 34.40 |
| Subject 2 | 59.89 | 0.65 | 75.52 | 54.01 | 85.02 |
| Subject 3 | 67.78 | 0.70 | 59.09 | 77.50 | 64.64 |
| Subject 4 | 62.84 | 0.54 | 61.82 | 62.24 | 48.43 |
| Subject 5 | 68.25 | 0.50 | 79.17 | 43.61 | 53.63 |
| Subject 6 | 68.33 | 0.65 | 70.61 | 72.88 | 66.04 |
| **Rest** | Acc | F1 | Spc | Sen/Rec | Prc |
| Subject 1 | 58.64 | 0.61 | 54.41 | 67.22 | 59.88 |
| Subject 2 | 61.61 | 0.50 | 61.61 | 25.81 | 46.23 |
| Subject 3 | 71.80 | 0.63 | 85.82 | 53.22 | 76.93 |
| Subject 4 | 57.27 | 0.46 | 82.42 | 36.62 | 72.39 |
| Subject 5 | 62.24 | 0.65 | 60.65 | 60.78 | 77.42 |
| Subject 6 | 76.91 | 0.70 | 83.21 | 59.33 | 82.03 |

**Table S2**. Performance metrics as assessed by comparison of level 3 annotation (from automated clusters based on frequencies between 1 Hz and 105 Hz) to manual annotations averaged over all 4 days for each subject. Acc = Accuracy; Spc = Specificity; Sen/Rec = Sensitivity/Recall; Prc = Precision. This table shows values also presented in Fig. S4. This performance is worse than the accuracy results reported with only frequencies up to 53 Hz as shown in the main text Table 1, particularly in the rest category.
